# Comparison of the Effects of Hugo's Point Massage and Play on IV-Line Placement Pain in Children: A Randomized Clinical Trial

**DOI:** 10.1155/2021/6612175

**Published:** 2021-05-26

**Authors:** Shahnaz Salawati Ghasemi, Mehdi Beyramijam, Fatemeh Yarahmadi, Taban Nematifard, Seyed Shahabeddin Bahrani, Masoomeh Khaleghverdi

**Affiliations:** ^1^Instructor, Nursing and Midwifery Faculty, Kurdistan University of Medical Sciences, Sanandaj, Iran; ^2^Hamadan University of Medical Sciences, Hamadan, Iran; ^3^Nursing, Nursing School of Boroujerd, Lorestan University of Medical Sciences, Khorramabad, Iran; ^4^Department of Nursing, University of Social Welfare and Rehabilitation, Tehran, Iran; ^5^Master of Medical-Surgical Nursing Education, Nursing and Midwifery School, Hamadan University of Medical Sciences, Hamadan, Iran; ^6^Department of Internal Medicine, School of Nursing and Midwifery, Hamadan University of Medical Sciences, Hamadan, Iran

## Abstract

Reduction of intravenous line placement pain is one of the most important nursing priorities in the pediatric wards. The present study was aimed at comparing the effect of Hugo's point massage and play on the severity of IV-line placement pain in hospitalized children aged 3–6 years in the pediatric ward. 72 children were selected and assigned randomly to three groups, i.e., control, play, and Hugo point massage. In the massage group, the middle angle between the first and second bones of the palm of the opposite hand was massaged, and the playgroup encouraged bubble-making play. The one-way analysis of variance (ANOVA) did not show a statistically significant difference between the mean IV-line placement pain in play, Hugo's point, and control groups before interventions (*p*=0.838; *p* > 0.05). However, the ANOVA test revealed a significant difference between the mean IV-line placement pain in play, Hugo's point, and control groups after the interventions (*p*=0.006; *p* < 0.05). The result of the post hoc Scheffe test also showed a statistically significant difference between the mean intensity of IV-line placement pain in both play therapy and Hugo's point massage groups (*p*=0.028; *p* < 0.05). Moreover, this test showed that the playgroup children felt less pain than Hugo's point and control group children. This study showed that, in comparison with Hugo's point massage, the play was a more effective way for reducing pain caused by IV-line placement in children, and pediatric nurses can play a significant role in reducing and managing children's pain by using it.

## 1. Introduction

IV-line placement pain is one of the most important nursing diagnoses in hospitalized children, which requires effective measures by nurses. Taking painful measures, such as venous port access and IV-line placement, is one of the commonest and most important factors causing pain in children [1]. Today, over 90% of hospitalized children undergo procedures of painful IV-line placement [2]. Most hospitalized children describe this procedure as the most stressful [3] and one of the most harmful aspects of hospitalization [4]. The pain of IV-line placement can stimulate the sympathetic system and cause changes in physiological indicators such as increased heart rate and respiration, decreased oxygen saturation, and increased blood pressure [5]. It can also lead to fear, nightmares, incontinence, and other physical and psychological adverse effects [6].

Among different age groups, children, specifically preschool children, are more sensitive to this painful procedure and usually respond more strongly [7, 8]. Also, the children have a poor picture of their body's borders and internal anatomy, and they often think that by inserting a needle and piercing the skin, the place will not be closed, and they will lose all their blood. Therefore, they may be more concerned about IV-line placement pain than the actual pain felt and may show a more severe reaction [9].

Any effort to decreasing pain can reduce the adverse effects of pain on various aspects of the children's development [10], greater patient satisfaction, and faster and easier access to blood vessels [11]. The Health Care Policy Organization considers the use of pharmacological and nonpharmacological methods alone or in combination with other methods as effective pain treatments [12]. In the meantime, the use of massage therapy in different parts of the patient's body, as one of the nonpharmacological methods, has been common for a long time [13].

According to the gate control theory of pain, skin stimulation through massage can stimulate the large fibers that carry nerve impulses to the spinal cord and, as a result, it keeps the pain gates closed and decreases pain feeling [14]. According to the Chinese, the body's vital energy, Chi, flows through channels, which are called meridians, and regulates the body's function, and blocking this energy in these channels disrupts and produces pain. By massaging certain points in the body, these channels can be accessed which is led to balance energy and improve pain [15]. One of these points is Hugo Point. Hugo is one of the pressure points of the large intestinal energy channel called large intestine 4 (LI4) and is located between the first and second metacarpal bones (between the thumb and forefinger) [16].

Play is another nonpharmacological method that can reduce pain in children. The play leads to the focus of the children on an external subject and separates him/her from the pain to maximize its adaptability. Therefore, the nurses can use it as part of the care, preparation, and cooperation of children in the procedures [17]. In most studies, the effects of play and Hugo's point massage have been investigated separately [6, 18, 19]. Also, most studies investigated the effects of bubble-making compared with other distraction methods such as using EMLA cream, regular breathing exercise, and touching the injection point [20–22], However, based on our knowledge, no study has been yet conducted for comparing the effects of bubble-making and Hugo's point massage on IV-line placement pain. Besides, studies conducted for the investigation of the effect of both methods on children's pain are contradictory. For example in the study of Sparks, the results showed that bubble-making did not have a significant effect on the pain of younger children [23]. Based on that, the present study was aimed to compare the effects of play and Hugo's point massage on pain caused by IV line placement in children in the age range from 3 to 6 years.

## 2. Materials and Methods

### 2.1. Trial Design

The present randomized clinical trial study was designed with three groups before and after the intervention in 2017–2018.

### 2.2. Participants

#### 2.2.1. Eligibility Criteria for Participants

The eligibility criteria for the participants are as follows: the children aged from 3 to 6 years, parental consent for the participation of the children in the study, the children who have mental, verbal, visual, and auditory abilities, the absence of any acute or chronic pain before IV-line placement, and the absence of first-degree relatives during IV-line placement. If the catheter was inserted into the skin more than once during the IV-line placement and the children did not cooperate in the intervention, they would be excluded from the study.

### 2.3. Study Setting

This study was performed in Besat Hospital in Sanandaj city.

### 2.4. Procedures

After teaching the necessary training on the Hugo point as well as on how to massage by an acupressure specialist, in the IV-line placement room, 3 minutes before the start of IV-line placement until its completion (fixation of angiocatheter), the middle angle between the first and second bones of the palm (the position between the thumb and forefinger) was massaged by researcher's thumb on a rotating basis and in the direction of clockwise movement.

The maximum time for the massage was one minute each time, and then the massage was stopped for 10 seconds, and this process continued again until the end of the IV-line placement. The duration of the IV-line placement (from inserting the needle to the fixation of the angiocatheter) was recorded by the researcher with the help of a stopwatch device.

For the playgroup in the IV-line placement room, a bubble maker was provided for the children, and the children were encouraged by the researcher to make the bubble 3 minutes before the IV-line placement until its completion (fixation of the angiocatheter) (in addition to routine procedures). The duration of the IV-line placement was recorded by the researcher using a stopwatch device.

**Figure 1:**
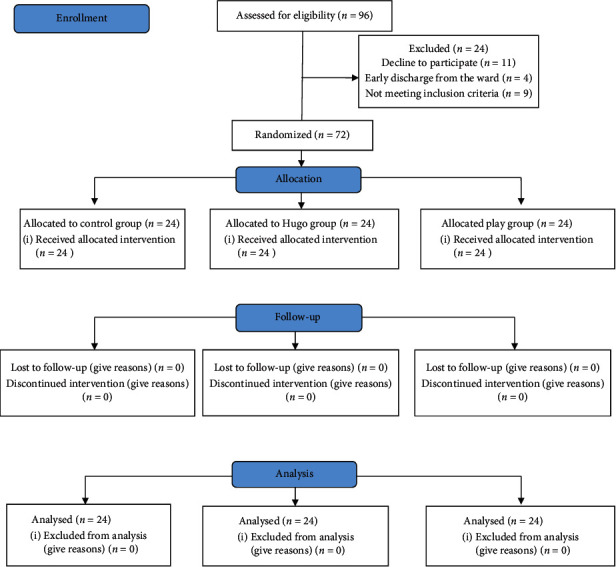
Consort flow diagram of the three study groups: control group; Hugo's point massage group, and playgroup.

For the control group in the IV-line placement room, only routine IV-line placement procedures (explaining the procedure and the need to do it for the parent and children, transferring the children to the IV-line placement room, washing hands, and preparing the equipment needed for IV insertion, choosing a vein and the appropriate place and the suitable angiocatheter, and putting the baby in the right position) was done for the children. The duration of the IV-line placement was recorded by the researcher with the help of a stopwatch device. In all three groups, the children's hands were used for IV-line placement and the severity of children's perceived pain was measured by the Wong and Baker scale before (to match the groups before the intervention) and immediately after IV placement. It should be noted that the IV-line placement procedure was performed by one of the nurses responsible for IV-line placement, using the same location for inserting the catheter (hands), same angiocatheter brands, and the same time (in the morning shift) for all three groups (Figure 1CONSORT flow diagram).

### 2.5. Outcomes

#### 2.5.1. Measures/Measurements

Data collection tools in this study included a children's demographic and clinical information questionnaire, a stopwatch device, and a Wong and Baker scale. The Wong and Baker scale is a tool for self-reporting pain intensity in children with the age of 3 years and above, developed by then in 1998 [9]. The scale consists of two parts as follows: a 0–10 numerical section for older children that can count and a section based on 6 different cartoon faces, at the top of the numerical section, for children who cannot count. In the 0–10 numerical section, the number that is mentioned by the children is indicative of his/her pain score. If a face scale is used, the face chosen by the children should be converted to scores of 10–0 so that the face at the left side is considered to be equal to 0, the second face is equal to 2, the third face is equal to 4, the fourth face is equal to 6, the fifth face is 8, and the sixth face is considered to be equal to 10. Zero is the painless condition and 10 is the most severe pain. In this study, since young children are not able to count, the Wong and Baker face scale was used in this study. The Persian version of this tool was used in the study of Nikfarid et al. [24] and the study of Alhani et al. [25]. In the study of Nikfarid, the reliability was obtained 82%. In this study, the interrater reliability method was used for determining the reliability of this tool and the reliability was obtained 87% by correlation coefficient test.

### 2.6. Sample Size

In this study, sampling was performed by the convenience sampling method from available samples. The sample size is based on the study of Maghsoudi et al. [26] with *S*1 = 1.1, *S*2 = 1.84, *x*1 = 1.7, and *x*2 = 3.81 with an assumption of the first type error of 5% and the second type error of 20% using the following formula. The sample size was estimated to be 20 people in each group, and 32 people were examined in each group to prevent possible falls (96 people).

(1) $$\begin{array}{c} n=\frac{{\left(s_{1}^{2}+s_{2}^{2}\right)}^{2}{\left(z_{1-\left(\alpha /2\right)}+z_{1-\beta }\right)}^{2}}{{\left(\bar{x_{1}}-\bar{x_{2}}\right)}^{2}}. \end{array}$$

### 2.7. Randomization and Allocation

The sample selection method was a randomized blocking method with 6-person blocks. In this way, all possible situations for people to be placed in each block were predicted. Then, all the blocks were numbered (12 6-person blocks), and then the blocks were randomly arranged. For example, two examples of blocks are as follows: ABCABC and ABBCABA…, where A was one of the intervention groups, B was the other intervention group, and C was the control group; that is, the first 12 people were placed in the study and groups from left to right, respectively, and it continued like this until the last person.

### 2.8. Ethical Consideration

After obtaining the approval of the Ethics Committee of the Research and Research Council of Kurdistan University of Medical Sciences and receiving the ethical code (IR.MUK.REC.1396/281) and Iranian Registry of Clinical Trials (code: IRCT20120215009014N302), the researcher performed the intervention in Hugo's point massage, play, and control groups after making the necessary arrangements and selecting patients with the criteria specifically parental consent for the participation of the children to enter the study. In the Hugo point massage group, after introducing and stating the objectives of the study and obtaining written consent, and attracting the cooperation of mother and children, the children with his/her the mother and the researcher went to the IV-line placement room.

### 2.9. Data Analysis

The one-way analysis of variance (ANOVA), post hoc Scheffe test, chi-square test (for determining the homogeneity of groups), Kolmogorov–Smirnov test (to define normal distribution), and descriptive statistics were used to analyze the data by SPSS software v.22.

## 3. Results

Out of 96 selected people, 24 were excluded from the study for various reasons (decline to participate, early discharge from the ward, and not meeting inclusion criteria), and finally, 72 people participated and were analyzed in the study. The mean age (standard deviation) of children in the Hugo massage, play, and control groups was 50.17 (9.756), 47.42 (9.297), and 50.96 (11.087) months, respectively, and in all three groups, gender segregation was equal. The cause of hospitalization of the majority of children in the Hugo massage group (25%), playgroup (20.8%), and control group (20.8%) was reported to be seizure caused by fever (see Table 1).

The one-way analysis of variance (ANOVA) did not show a statistically significant difference between the mean perceived pain in play, Hugo's point, and control groups before interventions (*p*=0.838; *p* > 0.05) (see Table 2).

However, the ANOVA test revealed a significant difference between the mean IV-line placement pain in play, Hugo's point, and control groups after the interventions (*p*=0.006; *p* < 0.05) (see Table 2). The result of the post hoc Scheffe test also showed a statistically significant difference between the mean intensity of IV-line placement pain in both play therapy and Hugo's point massage groups (*p*=0.028; *p* < 0.05). Moreover, this test showed that the playgroup children felt less pain than Hugo's point and control group children (see Table 2).

## 4. Discussion

The present study was aimed at comparing the effect of Hugo's point massage and play on the IV-line placement pain in young children. The results revealed a statistically significant difference between the mean IV-line placement pain in both the play and Hugo's point massage groups. In other words, the play had a greater impact on the IV-line placement pain in children. It seems that play in children is associated with the distraction of thoughts and employment of the five senses, which also reduces the pain caused by treatment. In the play, the children are actively involved, and he/she constantly employs the resources of concentration and reacts with stimuli of thought deviation, which leads to an increase in the children's pain tolerance threshold.

The obtained results also showed a significant difference between the intensities of IV-line placement pain in the groups of play and control. In line with the results of the present study, Maghsoudi et al. [26] stated that the use of distraction techniques such as play-dough and bubble-making was effective in reducing venepuncture pain in children. In the current study, the play was created with a bubble-making device. Also, the effect of Hugo's point massage was measured. But, in the study of “Maghsoudi et al., only play therapy with two different techniques has been studied.” Mousavi et al. [27], in a study conducted on the effectiveness of guided play on the severity of pain caused by changing the dressing of children's burn, showed that play before changing the dressing reduced the pain during the change of dressing. Although the procedure studied in the above study and the present study has not been the same, similar results have been obtained. In a review study conducted by Kapkin et al. [28] on the play therapy in hospitalized children, it was concluded that play therapy in children reduces the pain caused by invasive procedures and increases children's adaptation to the process of hospitalization and adaptation. In some studies that have examined the effects of thought distraction in age groups and various procedures, thought distraction has reduced the severity of pain, but statistical differences between groups have not been significant. In a study, Windich et al. [29] studied the effects of various distractions selected by children (such as bubble-making, exciting books, music, and video games) on the pain and fear of IV-line placement among 50 children and adolescents with cancer aged from 5 to 18 years. The results showed that the intervention group experienced decreased pain, but the groups were not significantly different [29].

Moreover, two Hugo's point massage and control groups were not significantly different in terms of mean IV-line placement pain. In most studies, the results were inconsistent with the results of the current study. In the study of Khosravan et al. [18], the results showed that the intensity of IV-line placement pain in infants was effectively affected by the massage of Hugo's point with and without ice. Probably the reason for the difference in results was the difference in the target group and the methodology of the two studies. In a study, Ecevit et al. examined the effect of acupuncture in preterm babies during minor painful procedures and found lower levels of pain in this group compared to control infants who received breast milk to reduce pain [30]. Also, the study of Rostami et al., by studying the effect of Hugo's point massage with ice on the intensity of IV-line placement pain in children with thalassemia, showed that the trial and control groups had a statistically significant difference in terms of the mean score of pain intensity. According to the authors, Hugo's point massage can reduce the intensity of IV-line placement pain in children with thalassemia [31]. In their study, only the effect of Hugo's massage with ice on pain intensity was measured in children with thalassemia, and the behavioral pain scale was used to measure the levels of pain.

Based on our research studies, due to the limited clinical trials in this field, no study compares the impact of Hugo's point massage and play on the intensity of IV-line placement pain in young children, which makes the comparison of the results difficult. Wang et al. [32] conducted a study on 300 children (age range of 8–9) and reported that the skin massage before the injection is as effective as play (watching a cartoon) in reducing the pain. In another study, Razaghi et al. [22] observed no significant difference between the intensity of pain in the bubble-making method and touch. It seems that the type of massage technique and the difference in massage points in different studies are the reason for the difference in results. Moreover, the different age groups studied are the causes of differences in results.

### 4.1. Practice Implications

As indicated by the results of the study, play therapy is a low-cost and cost-effective way that helps reduce IV-line placement pain in children.

### 4.2. Limitations

The effect of variables such as the children's mood and sociocultural background is of the limitations of this study; it can affect the child's experience, which was beyond the control of the researcher.

## 5. Conclusion

Based on the results of the present study, it can be said that bubble-making as a short-term, safe, cheap and attractive, and age-appropriate play can be effective in reducing IV-line placement pain in hospitalized children. The interventions used in this study can easily be used by nurses in other procedures and pediatric nurses can play a significant role in reducing and managing children's pain by using it. In the present study, bubble-making was used as a play. It is suggested that, in future studies, the effects of Hugo's point massage be compared with other games such as audio-visual equipment (i.e., cartoons, animation, and video games), squeezing rubber balls, Filippits distraction cards, or other games.

## Figures and Tables

**Table 1:** Demographic characteristics of the three groups.

Characteristics	Control		Hugo		Play		*p* value
	No.	Mean ± SD	No.	Mean ± SD	No.	Mean ± SD	0.446
Age (months)	24	50.96 ± 11.8	24	50.17 ± 9.75	24	47.42 ± 9.29	
Birth weight (kg)	24	15.56 ± 2.82	24	13.79 ± 2.47	24	13.76 ± 2.34	0.067
IV-line placement duration time (minutes)		3.2167 ± 00.67		3.37 ± 00.48		3.46 ± 00.60	0.600
	No.	Percent	No.	Percent	No.	Percent	

*Sex*							
Male	12	50.0	12	50.0	12	50.0	1.000
Female	12	50.0	12	50.0	12	50.0	

*Birthplace*							
Rural	11	45.8	12	50.0	11	45.8	0.946
Urban	13	54.2	12	50.0	13	54.2	

*Venous access location*							
Right hand	12	50.0	11	45.8	13	54.2	0.947
Left hand	9	37.5	10	41.7	8	33.3	
Right leg	2	8.3	3	12.5	2	8.3	
Left leg		4.2	24	100.0	1	4.2	

*Angiocatheter size*							
22	12	50.0	11	45.8	13	54.2	0.846
24	12	50.0	13	54.2	11	45.8	

*Taking painkillers*							
Yes	5	20.8	4	16.7	4	16.7	0.910
No	19	79.2	20	83.3	20	83.3	

*Number of hospitalizations*							
First time	7	29.2	8	33.3	11	45.8	0.568
Second time	14	58.3	12	50.0	10	41.7	
Third time	2	8.3	4	16.7	3	12.5	
Three times and more	1	4.2	—	—	—	—	

^*∗*^Homogeneity of groups was determined using chi-square tests.

**Table 2:** One-way analysis of variance (ANOVA) to compare the perceived pain intensity in study groups before and after IV-line placement.

	Group	Mean difference	Std. deviation	*F* value	*p* value	Difference ^*∗*^
Before	Control (a)	1.83	1.76	1.23	0.838	No difference
	Play (b)	2.00	1.56			
	Hugo's point (c)	1.67	1.40			

After	Control (a)	7.00	1.76	7.34	0.006	*a* > *b*
	Play (b)	5.12	2.27			*a* > *c*
	Hugo's point (c)	6.67	1.73			*c* > *b*

^*∗*^Post hoc Scheffe test was used to determine differences.

## Data Availability

The data used to support the findings of this study are available from the corresponding author upon request.
